# Symptoms similar to Kleine–Levin syndrome in a patient with basilar artery occlusion: a case report and literature review

**DOI:** 10.3389/fneur.2024.1293695

**Published:** 2024-08-30

**Authors:** Linghua Kong, Chunyan Li

**Affiliations:** Neurology Department, Binzhou Central Hospital, Binzhou Medical University, Binzhou, China

**Keywords:** Kleine–Levin syndrome, hypersomnia, pathogenesis, vascular disease, thrombectomy

## Abstract

**Introduction:**

Kleine–Levin syndrome (KLS) is a rare disorder characterized by recurrent periodic hypersomnia, cognitive disturbances, hyperphagia, and hypersexuality. Although many factors have been associated with its occurrence, little is known about treatment. Herein, we present a case of symptoms similar to KLS thought to be related to vascular occlusion disease.

**Case description:**

An 81-year-old woman was admitted to the hospital due to recurring episodes of disturbance of consciousness, cognitive disorder, and hyperphagia for 18 years. She was diagnosed with KLS and basilar artery occlusion. Endovascular and antithrombotic therapy was initiated, and her symptoms fully resolved within 2 weeks of treatment initiation.

**Conclusion:**

KLS has diverse clinical presentations and demonstrates variable therapeutic responses. Vascular disease or blood flow disorder may be one possible factor for this disease. This case underscores the need for further research into the etiology and pathogenesis of KLS to promote evidence-based approaches for its diagnosis and treatment.

## 1 Introduction

Kleine–Levin syndrome (KLS), also known as periodic hypersomnia syndrome, is a chronic sleep disorder characterized by recurrent episodes of hypersomnia, cognitive disturbances, hyperphagia, and hypersexuality. It mostly affects young individuals, with a reported prevalence of 1–5 cases per million across ages of 4 to 81 years. The clinical presentation of KLS is diverse, and the typical symptom triad involving hypersomnia, hyperphagia, and hypersexuality has been reported in only approximately 40% of the cases.

Many factors have been associated with the occurrence of KLS, such as infection, post-traumatic cerebral hemorrhage, multiple sclerosis, hydrocephalus, autoimmune encephalitis, and systemic tumors. Reported cases in young population appear to be primary while those in elders seem to be secondary to other neurological diseases. However, little is known about KLS treatment. Herein, we present a case with symptoms similar to KLS thought to be associated with vascular disease. The patient had basilar artery occlusion in her posterior circulation and fully recovered following thrombectomy from the underlying disease.

## 2 Case description

An 81-year-old woman was admitted to our hospital in June 2021 due to a recurrent consciousness disorder, with three previous episodes in 2003, 2004, and 2020. In all instances, she experienced the same symptoms: loss of consciousness for approximately 1–2 days, followed by cognitive disorder and hyperphagia. The most recent presentation occurred suddenly after dinner; she could not stand or walk, and her speech became inarticulate. After approximately 10 min, she lost consciousness without convulsions or urinary incontinence. She had a history of hypertension and cardiovascular disease for more than 20 years and had undergone cataract surgery on her right eye 10 years ago.

**Figure 1:**
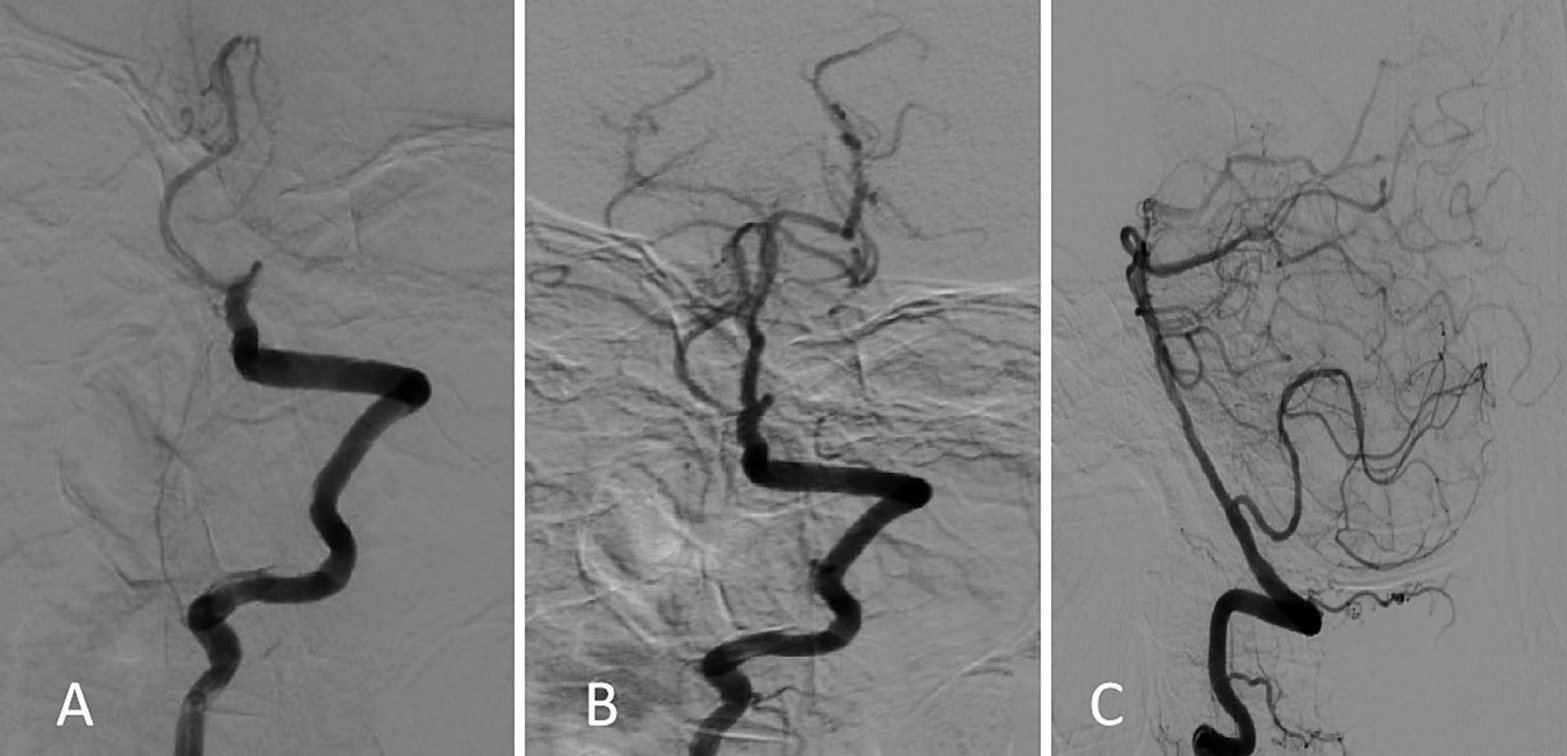
Digital subtraction angiography findings. **(A)** Initial image obtained on admission showing basilar artery occlusion and filling defects in the distal branches. **(B,C)** Follow-up images obtained after emergency bridging therapy using the “a direct aspiration first-pass technique” showing reestablished distal blood flow (mTICI 2b).

Physical examination revealed the following: Glasgow Coma Scale (GCS) 8, blood pressure 107/60 mmHg, bilateral pupils with an equal diameter of 2.5 mm with preserved reaction to light, no voluntary limb movement, positive hand and leg drop test, positive Babinski and Chaddock signs bilaterally, and national institutes of health stroke scale score (NIHSS) 35. Laboratory test findings were as follows: hemoglobin, 99 g/L, erythrocyte sedimentation rate, 23 mm/h; N-terminal pro-B-type natriuretic peptide, 7,863 pg/mL; antithyroglobulin antibody, 184.2 ng/mL; and anti-double-stranded deoxyribonucleic acid antibody, 227.82 IU/mL. Tumor markers and other biochemical parameter levels did not show significant abnormalities. Brain computed tomography showed no cerebral hemorrhage. As administration of low-dose recombinant tissue plasminogen activator (0.6 mg/kg) did not result in symptom improvement, emergency digital subtraction angiography was performed, which revealed basilar artery occlusion in the middle segment and blood perfusion defects in the distal segment (Figure 1).

After emergency bridging therapy using a direct aspiration first-pass technique (ADAPT), the basilar artery blood flow recovered to mTICI 2b. On the postoperative day 1, the patient’s GCS score was 8, the right pupil was irregular but reacted well to light, and the left pupil was round with a diameter of 2 mm and sensitive reaction to light. The remaining physical examinations showed results as before. After 5 days of combined therapy with an antiplatelet agent, statins, butylphthalide, aceglutamide, citicoline, and parenteral nutrition, her consciousness level improved (GCS 13), her speech became clear, and she was able to communicate. However, she still experienced episodes of disordered speech, bouts of delirium, hyperphagia, and could not recognize her family members. These symptoms occurred every night for several hours and resolved the next day. On physical examination, muscle strength was found to have improved to grade 4 in the upper limbs and grade 2 in the lower limbs. On the day 10, her consciousness completely recovered (GCS score 15) and muscle strength returned to nearly normal. Her symptoms significantly improved, and she fully recovered by day 14. She had four excessive sleepiness episodes lasting from 2 days to 2 weeks; her cognitive function and behavior were normal between the episodes. Hypersomnia could not be explained adequately by other problems of sleep, mental disorder, or substance abuse. The patient underwent a 24 h electroencephalogram examination during the first episode; however, as no epileptic wave appeared, her previous doctor did not administrate any antiepileptic medication.

## 3 Discussion

The first case of KLS was unofficially reported in the 17th century, while its first academic report was in 1925, when professor Kleine (1) first described the disease. In 1936, Professor Levin (2) systematically elaborated on its characteristics. The currently known term KLS was initially coined in 1942 by professors Critchley and Hoffman (3). The first Chinese case was reported in 1983 (4).

Most reported cases were sporadic, and familial cases were scarce. Some familial female cases suggested a close relationship of KLS with menstruation, because the first onsets in all affected female family members were after their menarches. This suggests that familial KLS may be associated with abnormal female hormone secretion. However, recurrent familial cases were not always related to menstruation or puerperium (5–7).

The largest number of KLS cases have been reported in the American population, with most cases in the age group of 10–20 years, with an average age of 15 years (8). Overall, the ages ranged from 4 to 80 years in male patients and 11 to 69 years in female patients. Based on available evidence, KLS appears to have male preponderance, accounting for 79.2% of all reported cases (9). Symptomatic episode periods have been reported to range from 3 to 21 days, with symptom-free intervals from 2 weeks to up to 17 years (9).

Although the triad of hypersomnia, hyperphagia, and hypersexuality is considered the typical clinical manifestation of KLS (10), less than half of the cases show this typical presentation. The majority present with the following symptoms: hypersomnia, confusion, apathy, insanity, or abnormal mental behavior (11, 12). The prevalence of depression in KLS is also high, ranging from 40.9 to 48% (13, 14); therefore, most early cases are first treated in psychiatric departments. In terms of diet, hyperphagia was predominant in Chinese cases; the patients could not stop eating voluntarily on account of their mental symptoms and required an intervention by family members to control the number of meals or suspend their eating activities in time.

Owing to the absence of specific diagnostic tests, the diagnosis of KLS is based on typical clinical manifestations. Several studies have confirmed that patients with KLS had no specific findings on computed tomography, magnetic resonance (MR), or single-photon emission computed tomography imaging (15, 16). Long-range electroencephalography monitoring showed slow diffuse background waves in bilateral frontotemporal lobes; however, these findings were not specific. Multiple sleep latency tests have indicated normal or slightly shortened mean latency, and numerous studies have reported no specific findings on polysomnography or abnormalities in sleep architecture and proportion (17, 18).

Diagnostic criteria for KLS have been diverse for a long time; different organizations and associations in Italy and India have published multiple versions of diagnostic recommendations for this disease. In 2014, the American Academy of Sleep Medicine published the third classification of sleep disorders for standardizing the diagnostic criteria (19). At present, the criteria from this classification have been adopted by most countries worldwide.

**Figure 2:**
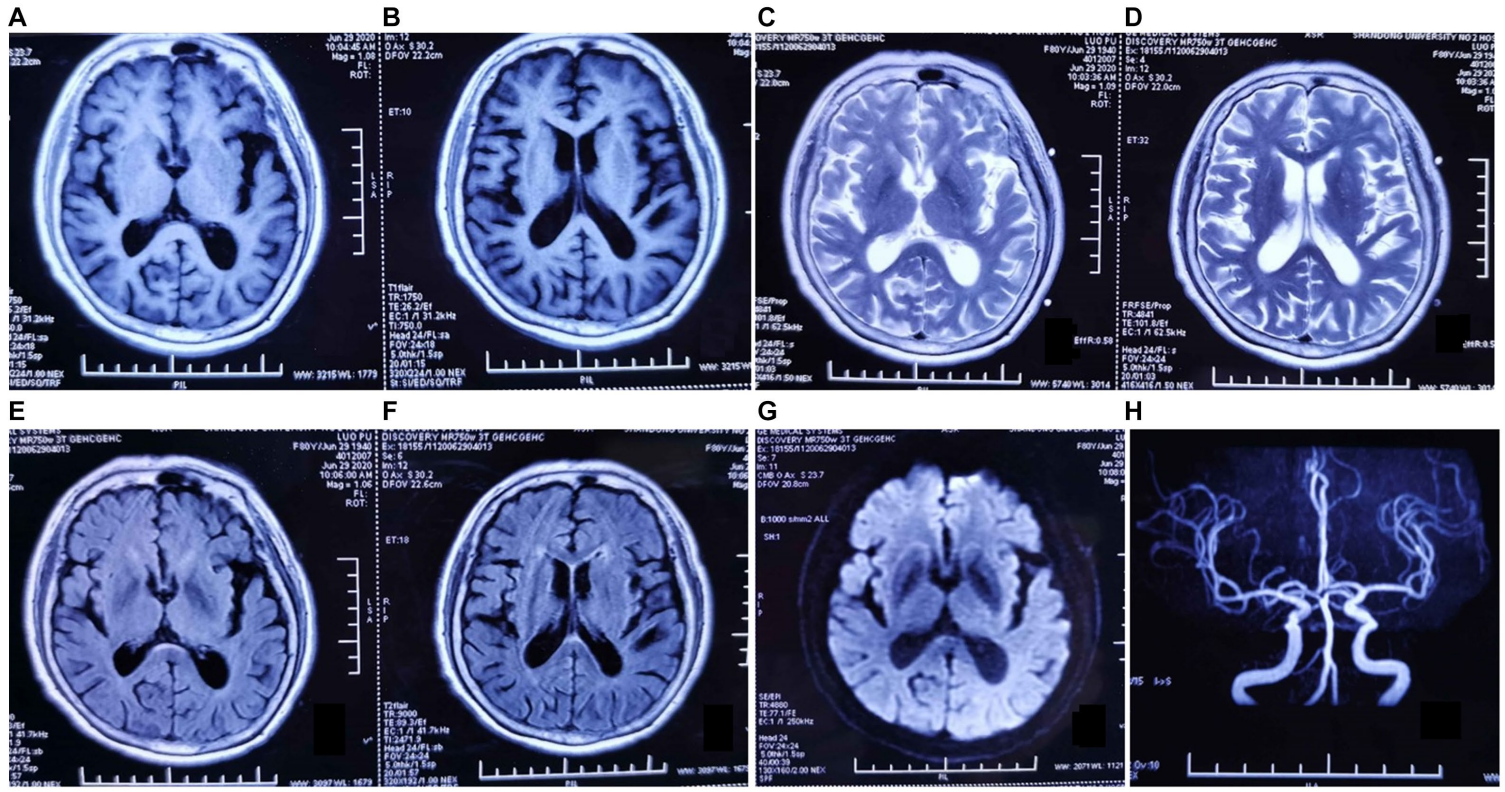
Magnetic resonance (MR) imaging findings dated June 2020. **(A–G)** Axial T1-weighted, T2-weighted, and fluid-attenuated inversion recovery MR images show mild demyelinating changes in the anterior horn of the lateral ventricle, abnormal punctate signals in the posterior limb of the internal capsule, and thickening of the cerebral dura mater. **(H)** MR angiography image showing no abnormalities in the large intracranial arteries.

**Figure 3:**
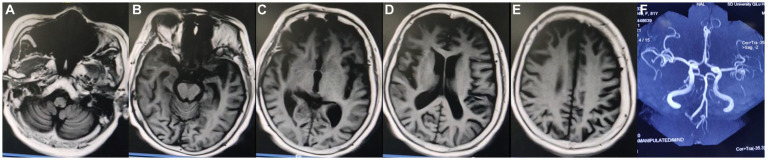
Magnetic resonance (MR) imaging findings dated June 2021. **(A–E)** Axial MR images showing mild atrophy of the cerebral cortex and thickening of the cerebral dura mater. **(F)** MR angiography image showing no abnormalities or stenosis in the large intracranial arteries.

Among more than 600 cases reported to date, approximately 10% were secondary to stroke, infection, post-traumatic cerebral hemorrhage, multiple sclerosis, hydrocephalus, autoimmune encephalitis, or systemic tumors. The etiology, pathogenesis, biological characteristics, and treatment of KLS remain poorly understood (20–22). Prior studies have suggested an association of KLS with autoimmune inflammation, physiological hormone level disorders, or genetic susceptibility (23, 24). Various treatment strategies have been attempted in the absence of a specific treatment. Amphetamine and methylphenidate were found to be effective in some cases, while lithium therapy resulted in the cessation of long attack episodes in others (25–28). In the current case, MR imaging performed in 2020 showed mild demyelinating changes and thickening of the cerebral dura, but large intracranial arteries were normal (Figure 2). MR imaging performed 1 year later, in June 2021, showed nearly the same findings as before. No abnormalities or stenosis in the large intracranial arteries were noted before this episode (Figure 3).

To date, little is known about the pathogenetic mechanism of KLS. Previous studies have indicated the involvement of the thalamus and pons in KLS pathogenesis. One study from 12 patients showed significantly lower functional connectivity on pontine-frontal eye field by functional MR imaging technology (29). Another study by Professor Mignot and his collaborators explored the interaction of genetic and environmental contributions to the pathophysiology; TRANK1-region variants were found to be significantly associated with KLS in 59 cases who reported birth difficulties. Differentially expressed proteins (DEPs) HLA-DQ/DR in cerebrospinal fluid were thought to be the most likely pathogenic site, while only one patient with sporadic KLS had a duplication in Xp22.31 (30).

When KLS occurs in a population suffering from other neurological diseases, it is referred to as secondary KLS. KLS seldom occurs with other diseases in young patients under 18 years of age, while older adults may suffer from some serious neurological diseases before their first KLS episode. To date, there are no clear diagnostic criteria for secondary KLS. According to the third classification of sleep disorders, secondary KLS appears to be a mixed complication of hypersomnia and other cognitive abnormalities. Although many factors have been associated with its occurrence, little is known about the underlying mechanism. Herein, we present a case of KLS thought to be related to vascular disease. Our patient presented with transient basilar arterial occlusion and blood flow disorders and reported full recovery following thrombectomy and antithrombotic therapy. This suggests a close relationship between KLS and vascular problems or blood flow disorders. In the present case, the episode frequency and duration, as well as onset age (over 80 years), differ from those of typical cases. Therefore, although we suggest that this case represents secondary KLS, we cannot exclude that it could just be KLS-like.

In conclusion, this case highlights the diversity in presentation and therapeutic response associated with KLS, and underscores the need for further research into its etiology and pathogenesis, while promoting evidence-based approaches for its diagnosis and treatment.

## Data availability statement

The original contributions presented in the study are included in the article/supplementary material, further inquiries can be directed to the corresponding author.

## Ethics statement

Written informed consent was obtained from the individual(s) for the publication of any potentially identifiable images or data included in this article.

## Author contributions

LK: Conceptualization, Writing – original draft, Writing – review & editing. CL: Data curation, Writing – original draft, Writing – review & editing.

## References

[1] Klein W. Periodische Schlafsucht. Monatsschr Psychiatr Neurol. (1925) 57:305–20. doi: 10.1159/000190427, (in German)

[2] Levin M. Narcolepsy (Gélineau’s syndrome) and other varieties of morbid somnolence. Arch Neurol Psychiatry. (1929) 22:1172–200. doi: 10.1001/archneurpsyc.1929.02220060069006

[3] Critchley M Hoffman HL. The syndrome of periodic somnolence and morbid hunger (Kleine–Levin syndrome). Br Med J. (1942) 1:137–9. doi: 10.1136/bmj.1.4230.137, PMID: 20784073 PMC2159883

[4] Zhong ZZ. Menstrual periodic hypersomnia. Int J Neurol Neurosurg. (1983) 6:316–7.

[5] Novitskaia AK. Sindrom Kleine–Levine (nabliudenie) [the Kleine–Levin syndrome (a case)]. Zh Nevropatol Psikhiatr Im S S Korsakova. (1991) 91:84–6. (in Russian) 1646549

[6] Nowak S Błaszczyk B. Zespół Kleine–Levine (opis przypadku) [Kleine–Levin syndrome (case report)]. Neurol Neurochir Pol. (1984) 18:179–81. (in Polish) 6592470

[7] Reddy MSS Sinha S Thippeswamy H Ganjekar S Chaturvedi SK. Kleine–Levin syndrome versus bipolar disorder not otherwise specified—diagnostic challenges. Asian J Psychiatry. (2017) 28:186–7. doi: 10.1016/j.ajp.2017.06.001 28784386

[8] Ambati A Hillary R Leu-Semenescu S Ollila HM Lin L During EH. Kleine–Levin syndrome is associated with birth difficulties and genetic variants in the TRANK1 gene loci. Proc Natl Acad Sci USA. (2021) 118:e2005753118. doi: 10.1073/pnas.2005753118, PMID: 33737391 PMC7999876

[9] Parrino L Ferri R Zucconi M Fanfulla F. Commentary from the Italian Association of Sleep Medicine on the AASM manual for the scoring of sleep and associated events: for debate and discussion. Sleep Med. (2009) 10:799–808. doi: 10.1016/j.sleep.2009.05.009 19564132

[10] Fernábdez J-M Lara I Gila L O'Neill A Tovar J Gimeno A. Disturbed hypothalamic-pituitary axis in idiopathic recurring hypersomnia syndrome. Acta Neurol Scand. (1990) 82:361–3. doi: 10.1111/j.1600-0404.1990.tb03317.x, PMID: 1963255

[11] Billiard M. Recurrent hypersomnias. Handb Clin Neurol. (2011) 99:815–23. doi: 10.1016/B978-0-444-52007-4.00008-4 21056229

[12] Afolabi-Brown O Mason TBA 2nd. Kleine–Levin syndrome. Paediatr Respir Rev. (2018) 25:9–13. doi: 10.1016/j.prrv.2016.12.004 28216256

[13] Minvielle S. Le syndrome de Kleine–Levin: une affection neurologique à symptomatologie psychiatrique [Klein–Levin syndrome:a neurological disease with psychiatric symptoms]. Encéphale. (2000) 26:71–4. (in French) 11064843

[14] Hu Y Wang JY Dong XS. Clinical characteristics of patients with recurrent hypersomnia. Chin Med J. (2017) 97:1236–9. doi: 10.3760/cma.j.issn.0376-2491.2017.16.011

[15] Engström M Hallböök T Szakacs A Karlsson T Landtblom AM. Functional magnetic resonance imaging in narcolepsy and the Kleine–Levin syndrome. Front Neurol. (2014) 5:105. doi: 10.3389/fneur.2014.00105, PMID: 25009530 PMC4069720

[16] Vigren P Engström M Landtblom AM. SPECT in the Kleine–Levin syndrome, a possible diagnostic and prognostic aid. Front Neurol. (2014) 5:178. doi: 10.3389/fneur.2014.00178, PMID: 25295028 PMC4172011

[17] Luo YW Yu H Yuan LH Zhu GX. A polysomnography study of Kleine–Levin syndrome in a single center. Chin Med J. (2016) 129:1565–8. doi: 10.4103/0366-6999.184476, PMID: 27364793 PMC4931263

[18] Huang YS Lin YH Guilleminault C. Polysomnography in Kleine–Levin syndrome. Neurology. (2008) 70:795–801. doi: 10.1212/01.wnl.0000304133.00875.2b 18316691

[19] American Academy of Sleep Medicine. International classification of sleep disorders: diagnostic and coding manual. 3rd ed. Westchester: American Academy of Sleep Medicine (2014).

[20] Mukaddes NM Alyanak B Kora ME Polvan O. The psychiatric symptomatology in Kleine–Levin syndrome. Child Psychiatry Hum Dev. (1999) 29:253–8. doi: 10.1023/A:1022621209493 10080967

[21] Vigren P Tisell A Engström M Karlsson T Leinhard Dahlqvist O Lundberg P. Low thalamic NAA-concentration corresponds to strong neural activation in working memory in Kleine–Levin syndrome. PLoS One. (2013) 8:e56279. doi: 10.1371/journal.pone.0056279, PMID: 23451038 PMC3581507

[22] Das S Gupta R Dhyani M Raghuvanshi S. Kleine–Levin syndrome: a case report and review of literature. Pediatr Neurol. (2014) 50:411–6. doi: 10.1016/j.pediatrneurol.2014.01.003 24630285

[23] Dauvilliers Y Mayer G Lecendreux M Neidhart E Peraita-Adrados R Sonka K. Kleine–Levin syndrome: an autoimmune hypothesis based on clinical and genetic analyses. Neurology. (2002) 59:1739–45. doi: 10.1212/01.WNL.0000036605.89977.D0 12473762

[24] Huang YS Guilleminault C Lin KL Hwang FM Liu FY Kung YP. Relationship between Kleine–Levin syndrome and upper respiratory infection in Taiwan. Sleep. (2012) 35:123–9. doi: 10.5665/sleep.1600, PMID: 22215926 PMC3242678

[25] Mignot EJ. A practical guide to the therapy of narcolepsy and hypersomnia syndromes. Neurotherapeutics. (2012) 9:739–52. doi: 10.1007/s13311-012-0150-9, PMID: 23065655 PMC3480574

[26] Paslakis G Träber F Roberz J Block W Jessen F. N-acetyl-aspartate (NAA) as a correlate of pharmacological treatment in psychiatric disorders: a systematic review. Eur Neuropsychopharmacol. (2014) 24:1659–75. doi: 10.1016/j.euroneuro.2014.06.004, PMID: 25130303

[27] de Oliveira MM Conti C Prado GF. Pharmacological treatment for Kleine–Levin syndrome. Cochrane Database Syst Rev. (2016) 2016:CD006685. doi: 10.1002/14651858.CD006685.pub4 27153153 PMC7386458

[28] Sveinsson O. A striking response to lithium in Kleine–Levin syndrome. Front Neurol. (2014) 5:33. doi: 10.3389/fneur.2014.00033, PMID: 24711803 PMC3968761

[29] Engström M Landtblom AM Karlsson T. New hypothesis on pontine-frontal eye field connectivity in Kleine–Levin syndrome. J Sleep Res. (2016) 25:716–9. doi: 10.1111/jsr.12428, PMID: 27230978

[30] Arnulf I Zeitzer JM File J Farber N Mignot E. Kleine–Levin syndrome: a systematic review of 186 cases in the literature. Brain. (2005) 128:2763–76. doi: 10.1093/brain/awh620, PMID: 16230322

